# The Management of Post-appendectomy Abscess in Children; A Historical Cohort Study and Update of the Literature

**DOI:** 10.3389/fped.2022.908485

**Published:** 2022-06-20

**Authors:** Paul van Amstel, Sarah-May M. L. The, Irene M. Mulder, Roel Bakx, Joep P. M. Derikx, Joost van Schuppen, Ralph de Vries, Martijn van der Kuip, Gerda W. Zijp, Jan Hein Allema, Taco S. Bijlsma, L. W. Ernest van Heurn, Ramon R. Gorter

**Affiliations:** ^1^Department of Pediatric Surgery, Emma Children’s Hospital, Vrije Universiteit Amsterdam, Amsterdam, Netherlands; ^2^Department of Pediatric Surgery, Emma Children’s Hospital, University of Amsterdam, Amsterdam, Netherlands; ^3^Amsterdam Reproduction & Development, Amsterdam, Netherlands; ^4^Amsterdam Gastroenterology Endocrinology Metabolism, Amsterdam, Netherlands; ^5^Department of Surgery, Noordwest Ziekenhuisgroep, Alkmaar, Netherlands; ^6^Department of Pediatric Surgery, Juliana Children’s Hospital/Haga-Hospital, The Hague, Netherlands; ^7^Department of Radiology, Emma Children’s Hospital, University of Amsterdam, Amsterdam, Netherlands; ^8^Department of Radiology, Emma Children’s Hospital, Vrije Universiteit Amsterdam, Amsterdam, Netherlands; ^9^Medical Library, Vrije Universiteit Amsterdam, Amsterdam, Netherlands; ^10^Department of Pediatric Infectious Diseases and Immunology, Amsterdam Infection & Immunity Institute, University of Amsterdam, Amsterdam, Netherlands; ^11^Department of Pediatric Infectious Diseases and Immunology, Amsterdam Infection & Immunity Institute, Vrije Universiteit Amsterdam, Amsterdam, Netherlands

**Keywords:** appendicitis, post-appendectomy abscess, children, invasive treatment, non-invasive treatment

## Abstract

**Introduction:**

Recent studies have shown that specific cases of post-appendectomy abscess (PAA) in children could be treated conservatively. However, due to the lack of high-quality evidence, choice of treatment still depends on preferences of the treating surgeon, leading to heterogeneity in clinical practice. Therefore, we aimed to provide an update of recent literature on the management of PAA in children and subsequently evaluate the outcomes of a large multicenter cohort of children treated for PAA.

**Methods:**

A literature search was performed in Pubmed and Embase, selecting all randomized controlled trials, prospective and retrospective cohort studies, and case series published from 2014 and onward and reporting on children (<18 years) treated for a PAA. Subsequently, a historical cohort study was performed, including all children (<18 years) treated for a radiologically confirmed PAA between 2014 and 2021 in a tertiary referral center and two large peripheral centers. Medical charts were reviewed to compare non-invasive (i.e., antibiotics) and invasive (i.e., drainage procedures) treatment strategies. Primary outcome was the success rate of treatment, defined as no need for further interventions related to PAA or its complications.

**Results:**

The search yielded 1,991 articles, of which three were included. Treatment success ranged between 69–88% and 56–100% for non-invasive and invasive strategies, respectively. Our multicenter cohort study included 70 children with a PAA, of which 29 (41%) were treated non-invasively and 41 (59%) invasively. In the non-invasive group, treatment was effective in 21 patients (72%) compared to 25 patients (61%) in the invasive group. Non-invasive treatment was effective in 100% of unifocal small (<3 cm) and 80% of unifocal medium size PAA (3–6 cm), but not effective for multiple abscesses.

**Conclusion:**

Non-invasive treatment of especially unifocal small and medium size (<6 cm) PAA in children seems to be safe and effective. Based on these results, a standardized treatment protocol was developed. Prospective validation of this step-up approach-based treatment protocol is recommended.

## Background

Appendectomy for acute appendicitis is considered a routine procedure with low morbidity. Although appendectomy is highly effective, post-operative complications remain common (1). One of the most encountered and feared complications after appendectomy is the development of an intra-abdominal abscess. Incidences of up to 24% have been reported, depending on the type of appendicitis and the surgical approach (2, 3). Traditionally, surgeons are trained with the dogma that pus should be evacuated from the body. Therefore, the standard treatment of a post-appendectomy abscess (PAA) consisted of drainage, either surgical or percutaneous with radiological guidance. However, several small cohort studies have reported that in the pediatric population specific cases of PAA could also be treated conservatively with or without antibiotics (4–6). In 2016, our research group published a paper on the outcomes of the treatment strategies for PAA. Based upon historical data, a step-up approach, reserving drainage procedures for those patients that are clinically ill and suspected of developing sepsis, was introduced in the Amsterdam University Medical Centres (UMC) (6). Despite this effort, choice of treatment depends on clinical, biochemical and radiological factors, but also on preferences of the treating surgeon. This in combination with the lack of high-quality evidence in current literature leads to large heterogeneity in current clinical practice (7).

Therefore, this study aimed to provide an update of the current literature by means of a systematic review of studies from 2014 and onward reporting on the treatment of PAA in the pediatric population. Subsequently, the outcome of a large cohort of children treated for PAA in a tertiary referral center and two large peripheral teaching hospitals were investigated to update the recommendations regarding the treatment for PAA in children.

## Materials and Methods

### Update of the Literature: Systematic Review

In 2016, our research group published a literature review of all studies from inception up to 2014 that reported on the management and outcomes of children with PAA. This review included six studies and found median (range) reported frequencies of persistent/recurrent abscess of 9% (0–30%) after non-invasive treatment, of 50% (0–100%) after percutaneous drainage, and of 24% (0–33%) after surgical drainage (6). In order to provide an update of the latest literature regarding the treatment of PAA, a systematic review of studies published from 2014 and onward was performed and reported according to the Preferred Reporting Items for Systematic reviews and Meta-analysis (PRISMA) guidelines (8).

#### Search Strategy

To identify relevant publications systematic searches in the bibliographic databases PubMed and Embase.com were conducted from inception to January 24, 2022, in collaboration with a medical information specialist (RV). The following terms were used (including synonyms and closely related words) as index terms or free-text words: “Appendectomy,” “Post-appendectomy,” “Abscess,” “Children.” The references of the identified articles were searched for relevant publications. Duplicate articles were excluded. All languages were accepted. The full search strategies for all databases can be found in Supplementary Appendix 1.

#### Study Selection

The systematic review included randomized controlled trials, prospective or historical cohort studies and case series that were published from 2014 and onward. Case reports, expert opinions, conference abstracts, letters to the editor, and articles written in other languages than English, Dutch, German, and French were excluded. All studies reporting on children (<18 years) treated for PAA were included. Both studies that report on non-invasive treatment strategies consisting of clinical/outpatient monitoring with or without antibiotics (IV/oral) and studies reporting on invasive treatment strategies consisting of percutaneous drainage procedures with or without IV or oral antibiotics or surgical procedures (laparoscopic or open) with or without IV or oral antibiotics, were eligible for inclusion. PAA was defined as a radiologically confirmed accumulation of purulent fluid in a walled-off space within the abdominal cavity after open or laparoscopic appendectomy, accompanied by clinical and biochemical signs of infection. Only studies that reported our primary outcome treatment success, defined as no need for additional interventions related to the treatment of PAA or its complications within 90 days after initial treatment of PAA, were considered for inclusion. Secondary outcomes that were investigated were the number of additional interventions, length of hospital stay, number of readmissions, number of imaging studies, and number of outpatient visits.

#### Assessment of Methodological Quality and Data Collection

Included articles were evaluated by two independent reviewers (PA, ST) for methodological quality. Methodological quality of the studies was assessed using ROBINS-I (9). Two independent reviewers screened potential eligible articles based on title and abstract and subsequently full text articles were assessed for eligibility. Disagreements were resolved in consensus (PA, ST). Data regarding the primary and secondary outcomes of this review were collected by two authors (PA, ST) independently.

#### Statistical Analysis

Only descriptive statistics were performed using IBM SPSS statistics version 26 (IBM SPSS 26.0, Armonk, NY, United States). Methodological heterogeneity was expected to be substantial and therefore the results were not pooled in a meta-analysis.

### Multicenter Historical Cohort Study

For the second part of the study, a multicenter historical cohort study was conducted in which all patients (<18 years old) treated for PAA at the Amsterdam UMC, Northwest Hospital Alkmaar (NWZ), and Juliana Children’s Hospital/Haga-Hospital (JKZ) between the 1st of January 2014 and 31st of December 2021 were included. PAA was defined as a radiologically confirmed accumulation of purulent fluid in a walled-off space within the abdominal cavity after open or laparoscopic appendectomy, accompanied by clinical and biochemical signs of infection. Patients that developed an intra-abdominal abscess after non-operative treatment for the suspicion of simple or complex appendicitis were excluded. Furthermore, patients treated for a suspicion of a PAA but without radiological confirmation were excluded from this study. ICD codes of acute appendicitis, acute abdomen, and intra-abdominal abscess were used for the identification of eligible patients.

The study protocol was reviewed by the Medical Ethics Review Committee of all participating centers. All three Medical Ethics Review Committees confirmed that the Dutch Medical Research Involving Human Subjects Act does not apply and therefore the need for complete ethical review was waived (Medical Ethics Review Committee reference number: W20_485#20.537). Identified patients received a letter with information about the study and their rights to object to the use of their data. Patients that objected against the use of their data were excluded from this study.

#### Data Extraction and Analysis

Patient records were reviewed and data were stored in an online database (Castor EDC). An online extraction form was used to review the medical charts and collect data regarding the initial appendectomy, baseline characteristics, treatment of PAA, and follow-up. Definitions of appendicitis severity, secondary interventions, and the most frequently encountered complications can be found in Supplementary Appendix 2. Outcomes of non-invasive and invasive treatment strategies were analyzed and described for patients with a small PAA (<3 cm), a moderate PAA (3–6 cm), a large PAA (>6 cm), and those with multiple PAAs. In one of the peripheral teaching hospitals non-invasive treatment strategies were preferred, while the other teaching hospital preferred invasive drainage procedures. Therefore, a subgroup analysis was performed, describing the outcomes for the participating centers individually.

#### Outcome Measures

Primary outcome measure was the success rate of the treatment strategies, which was defined as no need for additional interventions related to the treatment of PAA or its complications within 90 days after initial treatment of PAA. Additional interventions were defined as the need for additional antibiotics and/or percutaneous drainage procedures and/or surgical drainage procedures. Complications were classified according to the Clavien-Dindo classification. Clavien-Dindo grade 1 was defined as any deviation from the normal course after intervention without the need for pharmacological treatment or a surgical/radiological intervention, grade 2 as complication requiring pharmacological treatment, grade 3 as complication requiring surgical/radiological intervention, grade 4 as a life-threatening complication, and grade 5 as a complication resulting in death of a patient (10). Secondary outcomes were the initial and total length of hospital stay, readmission rate related to PAA treatment, number of imaging studies, and number of outpatient visits related to PAA treatment or its complications.

#### Statistical Analysis

For the primary and secondary outcomes, only descriptive statistics were performed. Continuous variables are presented as mean with standard deviation or median with interquartile ranges according to their distribution. Student’s *T*-tests and Mann–Whitney *U*-test were performed for the comparison of normally and non-normally distributed continuous variables, respectively, and the Chi square or Fisher exact test for categorical variables. Subsequently, a subgroup analysis was performed in which the primary outcome was described for the participating centers individually. A *p*-value < 0.05 was considered statistically significant. All statistics were performed using IBM SPSS statistics version 26 (IBM SPSS 26.0, Armonk, NY, United States).

## Results

### Update of the Literature: Systematic Review

**FIGURE 1:**
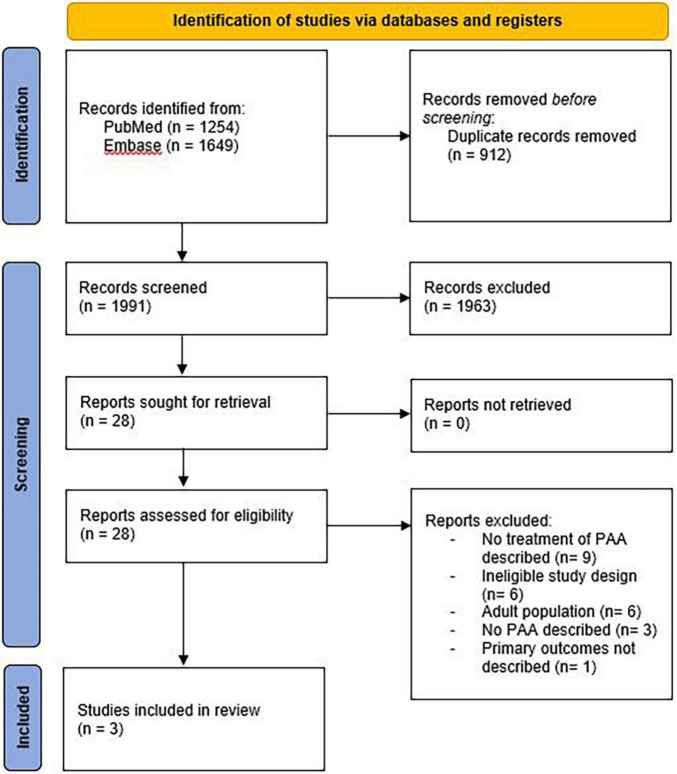
Preferred reporting items for systematic reviews and meta-analysis (PRISMA) flowchart.

The literature search generated 2,903 references; 1254 in PubMed and 1649 in Embase.com. After removal of duplicates, 1,991 articles were screened for title and abstract. After screening, 28 articles were assessed for full text, of which 25 were excluded. A flowchart of the study selection can be found in Figure 1. Study designs of the three included studies were one prospective cohort study and two historical cohort studies (6, 11, 12). Risk of bias was assessed as moderate in the prospective cohort and serious in both historical cohort studies. Results of the systematic review are displayed in Table 1.

**TABLE 1:** Results of the systematic review.

Author (year)	Study design	Intervention	Treatment success	Additional intervention	Complication
Svetanoff et al. (12)	Prospective cohort	AB: 8	7 (88%)	1 surgery	Small bowel obstruction: 1
		PD: 22	21 (95%)	1 PD/SD	Recurrent abscess: 1
Gorter et al. (6)	Historical cohort	AB: 13	9 (69%)	4 PD, 1 SD	Persistent/recurrent abscess: 4
		PD: 9	5 (56%)	2 PD, 4 SD	Persistent/recurrent abscess: 3
					Iatrogenic bowel perforation: 1
					Secondary bowel obstruction: 2
					Splenic hemorrhage: 1
					Superficial site infection: 1
					SBO due to adhesions: 1
		SD: 3	3 (100%)	–	–
Emil et al. (11)	Historical cohort	AB: 21	18 (86%)	2 PD, 1 SD	Persistent/recurrent abscess: 3
		PD: 19	18 (95%)	1 SD	Persistent/recurrent abscess: 1
		SD: 2	2 (100%)	–	–

*AB, antibiotics; PD, percutaneous drainage; SD, surgical drainage.*

In the prospective cohort study reporting on 30 children with PAA, the implementation of a treatment algorithm for PAA was evaluated (12). This algorithm recommends non-invasive treatment with antibiotics for PAA smaller than 20 cm^2^ (i.e., diameter <2,5 cm) and aspiration and/or percutaneous drainage for PAA larger than 20 cm^2^. Seven patients with a small PAA were treated with antibiotics, of which only one underwent a secondary intervention due to small bowel obstruction. In the group of patients with a large abscess, 18 of 19 were successfully treated with aspiration or percutaneous drainage of the abscess. One patient underwent a secondary intervention due to persistent abscess.

In both historical cohort studies three different interventions for the treatment of PAA were described: antibiotics (IV or oral), percutaneous drainage, and surgical drainage. The study of Emil et al. reports on 42 patients, of which 21 were initially treated non-invasively with antibiotics; 19 underwent imaging-guided percutaneous drainage, and two underwent surgical drainage. Treatment success rate was 18/21 (86%) in the group of patients that were treated with antibiotics. Two patients underwent percutaneous drainage and one patient surgical drainage for persistent abscess. Percutaneous drainage was successful in 18 of 19 patients (95%). One patient proceeded to surgical drainage of a persistent abscess. Initial surgical drainage was successful in all patients. Readmissions were more frequent in the non-invasive treatment group (33% of patients) compared to the drainage group (10%). Length of hospital stay was significantly longer after invasive treatment (mean of 15.9 ± 5.4 vs. 12.2 ± 4.6 days) (11).

The study by our own research group reports on 25 patients, of which 13 were initially treated non-invasively with oral or IV antibiotics, 9 with percutaneous drainage, and 3 patients underwent surgical drainage. Treatment was successful in 9 of 13 patients (69%) that were treated non-invasively. All four patients were subsequently treated for persistent/recurrent abscess with percutaneous drainage, which was successful in 3 of 4 patients. Initial percutaneous drainage was successful in 5 of 9 patients (56%), and surgical drainage in all three patients. In the non-invasive group a total of five interventions were needed (four percutaneous and one surgical) compared to 20 in the invasive group (14 percutaneous and six surgical). Patients treated non-invasively underwent a median of 3 ultrasounds per patient compared to 4 ultrasounds per patient in the drainage group. Length of hospital stay was significantly longer for the invasive treatment group [median (range) of 17 (1–42) days vs. 7 (1–22) days in the non-invasive group]. No differences between groups were found regarding the number of outpatient check-ups (6).

#### Excluded Studies

After full text assessment, 25 studies were excluded. Reasons for exclusion are displayed in Figure 1. One of the excluded studies did describe the treatment of PAA in children, but did not report the effectiveness of treatment strategies and was therefore excluded (13). In this study, patients were treated either with aspiration/drainage procedures with administration of antibiotics or non-invasively with antibiotics alone. Aim of the study was to compare those patients that were treated with oral vs. those treated with IV antibiotics. Results showed that oral antibiotics offer equivalent outcomes to IV antibiotics, but with a shorter length of hospital stay, less hospital visits, and without the morbidity of PICC-line placement (13).

### Multicenter Historical Cohort Study

During the study period, 1,346 patients underwent appendectomy in the participating centers. Of these, 70 patients (5%; 95% CI: 4–7%) developed a PAA. Baseline characteristics are displayed in Table 2. Laparoscopic appendectomy was performed in 50 patients (71%), open appendectomy in 18 patients (26%), and in two patients (3%) data regarding the surgical approach was missing. In 10 patients appendicitis severity was classified as simple, in 58 patients as complex, and in two patients appendicitis severity could not be determined due to missing surgery reports and/or histopathological examination reports. Of the patients with simple appendicitis, one developed a PAA smaller than 3 cm, two a PAA between 3 and 6 cm, and three a PAA larger than 6 cm. Of those with complex appendicitis, seven developed a PAA smaller than 3 cm, 21 a PAA between 3 and 6 cm, 15 a PAA larger than 6 cm, 14 developed multiple PAAs, and the size of PAA was unknown in one patient. PAA was diagnosed after a median of 9 days (IQR: 6–13 days). Method of imaging was ultrasonography in 52 patients (74%), Magnetic Resonance Imaging (MRI) in 16 patients (23%), and Computed Tomography (CT) in two patients (3%). In 29 patients an initial non-invasive treatment strategy for PAA was preferred. Of these 29 patients, 23 were treated with IV antibiotics, one patient was admitted with oral antibiotics, two patients were treated with oral antibiotics without admission to the pediatric ward, and three patients did not receive any antibiotics and were monitored for signs of clinical deterioration during outpatient visits. In 41 patients an invasive treatment strategy was preferred. Of these, 32 patients underwent imaging guided percutaneous drainage, in six patients the PAA was drained laparoscopically, in two patients open surgical drainage was performed, and in one patient the initial laparoscopic drainage procedure was converted to open drainage. Reasons for invasive treatment were large size of PAA and/or multiple PAA in 23 patients, unstable clinical condition with signs of sepsis in seven patients, suspicion of stump leakage in two patients and a persistent intra-abdominal fecalith in one patient. The reason for invasive treatment was not reported in eight patients. The initial non-invasive treatment and invasive treatment groups were comparable regarding the temperature at diagnosis of PAA, leukocytes, and CRP levels. In the group of patients with PAA smaller than 3 cm, most patients were treated non-invasively (88%), whereas in the group of patients with a PAA larger than 6 cm and those with multiple PAAs invasive treatment was preferred (84 and 79% respectively).

**TABLE 2:** Baseline characteristics.

	Non-invasive (*n* = 29)	Invasive (*n* = 41)	*p*-value
Male gender	16 (44.8%)	24 (58.5%)	0.258
Age (years)^	9.3 ± 3.8	10.2 ± 4.0	0.338
Appendicitis severity			1.0
- Simple	4 (14%)	6 (15%)	
- Complex	25 (86%)	33 (80%)	
- *Missing*	*0*	*2*	
Surgical approach			0.045
- Laparoscopic	17 (59%)	33 (80%)	
- Open	11 (38%)	7 (17%)	
- *Missing*	*1*	*1*	
Time after appendectomy (days)*	9 (6.5–13)	9 (6–12.5)	0.957
Temperature at diagnosis PAA	37.6 (37.1–38.3)	37.7 (37.1–38.6)	0.496
Leukocytes at diagnosis PAA (x10^9^/L)*	17.3 (14.0–24.3)	18.0 (15.0–22.2)	0.760
CRP at diagnosis PAA (g/dL)*	105 (74–191)	154 (83–216)	0.458
PAA size			<0.001
- Small (< 3 cm)	7 (24%)	1 (2%)	
- Medium (3–6 cm)	15 (52%)	12 (29%)	
- Large (>6 cm)	3 (10%)	16 (39%)	
- Multiple	3 (10%)	11 (27%)	
- *Missing*	*1*	*1*	
PAA location			0.155
- RLQ	14 (48%)	15 (37%)	
- RUQ	4 (14%)	2 (5%)	
- Douglas space	6 (21%)	13 (32%)	
- LLQ	1 (3%)	–	
- LUQ	1 (3%)	–	
- Multiple	3 (10%)	11 (27%)	

* Median (IQR).

*^∧^Mean and standard deviation.*

#### Primary Outcome

The success rate of both treatment strategies is displayed in Table 3. Treatment was successful in 21 of 29 patients (72%) that were treated non-invasively. In the group of patients with a PAA smaller than 3 cm, non-invasive treatment was successful in all patients and in those patients with a PAA between 3 and 6 cm non-invasive treatment was successful in 80% of patients. In the group of patients with PAA >6 cm and in those with multiple PAAs the success rate of non-invasive treatment was 67 and 0% respectively.

**TABLE 3:** Treatment success rate, secondary interventions, and complications.

	Non-invasive (*n* = 29)	Invasive (*n* = 41)
**Treatment success (overall)**		
- Small (<3 cm)	7/7 (100%)	1/1 (100%)
- Medium (3–6 cm)	12/15 (80%)	8/12 (67%)
- Large (>6 cm)	2/3 (67%)	10/16 (63%)
- Multiple	0/3 (0%)	7/11 (64%)
- Unknown size	0/1 (0%)	1/1 (100%)
Persistent/recurrent abscess	7 (24%)	9 (22%)
**Secondary interventions**		
- Percutaneous drainage	5	5
- Open drainage	1	2
- Laparoscopic drainage	1	–
- Oral antibiotics (outpatient clinic)	–	1
- IV antibiotics + admission	–	1
- Intervention for other complication	1	5
**Other complications**		
- Ileus	–	3 (2x CD-III, 1x CD-II
- Surgical Site Infection	–	1 (CD-III)
- Incisional hernia	–	1 (CD-III)
- CVC related infection	–	1 (CD-II)
- Pleural empyema	1 (CD-III)	–
- Fistula	1 (CD-II)	–
- Suspicion of bowel perforation	–	1 (CD-III)
- Fever	–	1 (CD-II)
- Vaginal blood loss after transrectal drainage procedure	–	1 (CD-I)

*Data is displayed as count (percentage).*

*CD, clavien-dindo; CVC, central venous catheter; IV, intravenous.*

Seven patients underwent a secondary intervention due to persistent or recurrent abscess. Of these, five underwent imaging guided percutaneous drainage, one was treated by laparoscopic drainage, and one by open drainage. Another patient was diagnosed with a subhepatic PAA that was successfully treated with antibiotics, but this patient developed right-sided pleural empyema that was treated with two thoracic drainage procedures. All secondary interventions were successful and no further drainage procedures or other secondary interventions were needed in this patient group.

Invasive treatment was successful in 27 of 41 patients (66%). In all patients with a PAA smaller than 3 cm, treatment was successful. In the group of patients with a PAA between 3 and 6 cm, in those with PAA larger than 6 cm, and in the group of patients with multiple PAAs, treatment success was 67%, 63%, and 64% respectively. Nine patients that were treated with an invasive treatment strategy underwent secondary treatment for a persistent or recurrent abscess, of which five underwent a secondary imaging guided percutaneous drainage and two patients were treated with open drainage. Two patients with a persistent or recurrent abscess were treated with antibiotics, of which one was readmitted with IV antibiotics, and one was treated with oral antibiotics without readmission. Five other patients underwent secondary surgery due to complications of PAA treatment. Two of these were treated for small bowel obstruction caused by adhesions around the old drain track, one developed a surgical site infection after PAA treatment and underwent surgical drainage of a wound abscess, and one patient underwent secondary surgery due to a suspicion of bowel perforation after imaging guided percutaneous drainage. During surgery no perforation was found, however, a persistent abscess was surgically drained. Lastly, one patient underwent surgical repair of an incisional hernia 3 months after surgical drainage of a PAA.

#### Subgroup Analysis: Treatment Success for Each Participating Center

In Table 4 the primary outcome, success rate of treatment strategies, is displayed for each participating center individually. Non-invasive treatment strategies were preferred in teaching hospital A, whereas teaching hospital B preferred invasive drainage procedures. Of specific interest is the group of patients with PAA between 3 and 6 cm. In teaching hospital A, 10 of 11 patients with 3–6 cm PAA were treated with antibiotics, which was successful in 90% of these patients. In teaching hospital B, percutaneous or surgical drainage was performed in seven of eight patients with 3–6 cm PAA. Treatment was successful in six of seven patients (86%) that underwent drainage procedures for 3–6 cm PAA.

**TABLE 4:** Primary outcome divided by participating center.

	Non-invasive (*n* = 7)	Invasive (*n* = 19)
**Treatment success (Tertiary referral center)**		
- Small (<3 cm)	2/2 (100%)	–
- Medium (3–6 cm)	3/4 (75%)	2/4 (50%)
- Large (>6 cm)	1/1 (100%)	5/9 (56%)
- Multiple	–	3/6 (50%)

	**Non-invasive (*n* = 19)**	**Invasive (*n* = 4)**

**Treatment success (Teaching hospital A)**		
- Small (<3 cm)	4/4 (100%)	–
- Medium (3–6 cm)	9/10 (90%)	0/1 (0%)
- Large (>6 cm)	1/2 (50%)	0/1 (0%)
- Multiple	0/3 (0%)	1/1 (100%)
- Unknown size	–	1/1 (100%)

	**Non-invasive (*n* = 3)**	**Invasive (*n* = 18)**

**Treatment success (Teaching hospital B)**		
- Small (<3 cm)	1/1 (100%)	1/1 (100%)
- Medium (3–6 cm)	0/1 (0%)	6/7 (86%)
- Large (>6 cm)	–	5/6 (83%)
- Multiple	–	3/4 (75%)
- Unknown size	0/1 (0%)	–

*Data is displayed as count (percentage of total).*

#### Secondary Outcomes

The results regarding the length of hospital stay, number of imaging studies, number of readmissions, and the number of outpatient visits are displayed in Table 5. Initial and total length of hospital stay were somewhat longer for the group of patients that were treated invasively compared to those that were treated non-invasively. Furthermore, these patients underwent more imaging studies and were more frequently readmitted than the non-invasively treated patients. Ultrasound was most frequently used for follow-up of patients.

**TABLE 5:** Secondary outcomes.

	Non-invasive (*n* = 29)	Invasive (*n* = 41)
Initial length of stay (day)	7 (0–17)	8 (4–31)
Total length of stay (day)	7 (0–17)	9 (4–31)
No. of imaging studies per patient	3 (2–4)	4 (3–6)
No. of ultrasounds per patient	3 (0–5)	4 (0–14)
No. of MRIs per patient	0 (0–3)	0 (0–3)
No. of CT-scans per patient	0 (0–1)	0 (0–2)
No. of readmitted patients*	1	7
Number of readmissions*	1	9
Outpatient visits	2 (0–7)	1 (0–9)
Telephone check-up	0 (0–4)	0 (0–4)

Data is displayed as median (range). *Data is displayed as count.

## Discussion

Our results show that non-invasive treatment of children with a PAA can be successful, especially for patients in a stable clinical condition without signs of sepsis that are diagnosed with a PAA smaller than 6 cm. In our multicenter historical cohort, non-invasive treatment of PAA smaller than 6 cm without signs of sepsis was successful in 19 of 22 patients (86%). Our systematic review of the literature shows that since 2014 only few new studies have been published on this topic. Results of these studies are in line with our study, confirming that small to moderate sized abscesses (<6 cm) can be successfully treated non-invasively.

Literature regarding the management of PAA is scarce and high-quality evidence derived from randomized controlled trials or large standardized prospective studies is lacking. Thus far, only one small prospective cohort study and few historical cohort studies have been published. These studies report that treatment success rates range between 69 and 100% after non-invasive treatment, between 0 and 100% after percutaneous drainage, and between 36 and 100% after surgical drainage (4–6, 11, 12, 14–17). Our study reports comparable results, since non-invasive treatment was successful in 21 of 29 patients (72%) and invasive treatment in 27 of 41 patients (66%).

Although favorable results of non-operative treatment have been reported by previous studies, some surgeons are still reluctant to treat children with a PAA non-invasively, as high-quality evidence is lacking. Moreover, historically surgeons were trained with the dogma that intra-abdominal pus should be evacuated from the body. This leads to large heterogeneity in treatment of PAA, which was also demonstrated in our study, especially for the group of patients with moderate size (3–6 cm) PAA. In the entire cohort, 15 patients with moderate size PAA were treated non-invasively and 12 invasively. These treatment groups should be compared with caution due to possible confounding by indication. Treatment decisions are based on multiple factors, including surgeons’ preference, clinical condition of the patient, location of the abscess, and most importantly the availability of a skilled (pediatric) interventional radiologist. Major strength of this study, however, is the participation of two large peripheral teaching hospitals with different philosophies toward treatment of PAA, which improves comparability of treatment strategies. Focusing on the group of patients with a 3–6 cm PAA, treatment success of antibiotic treatment was 90% in the teaching hospital that preferred non-invasive treatment strategies, compared to 86% for drainage procedures in the teaching hospital that preferred invasive treatment strategies. Therefore, non-invasive treatment seems to be at least equally successful compared to invasive drainage procedures in this group of patients. Although very effective, drainage of PAA is an invasive procedure that is associated with significant morbidity, a longer length of hospital stay and higher number of imaging studies compared to non-invasive treatment (4, 6, 11). Moreover, percutaneous drainage of PAA in children requires general anesthesia.

The treatment of PAA smaller than 3 cm is less subject of debate in children. Although historically, especially in adults, invasive treatment procedures for PAA were preferred, several more recent studies have reported promising results of non-operative treatment of small PAA with treatment successes ranging between 84 and 100% (6, 12, 18–20). This is in line with the results of our multicenter cohort, in which all patients with PAA smaller than 3 cm were successfully treated with a non-invasive treatment strategy.

Even in some cases of large (>6 cm) PAA, non-invasive treatment strategies have been reported to be successful (6). In our study three patients with a large PAA were treated non-invasively, which was successful in two of them. Although non-invasive treatment is potentially effective in some cases of large PAA, invasive drainage procedures that have been reported to be equally successful are usually preferred for large PAA (6, 21). As reported in our study, in the majority of patients (16 of 19 patients) drainage procedures were performed. The decision to the preference for invasive treatment strategies in patients with large PAA is mostly based on expert opinion and the idea that resorption of the abscess could be time consuming when treated non-invasively, potentially leading to longer length of hospital stay, longer duration of antibiotic treatment and higher number of imaging studies in this patient group.

In line with the treatment of large PAAs, invasive treatment strategies are preferred in the majority of patients presenting with multiple PAAs. In our study, 11 of 14 patients with multiple PAAs underwent invasive drainage procedures. Moreover, non-invasive treatment was not successful in the three patients that underwent initial non-invasive treatment for multiple PAAs. Therefore, percutaneous drainage is recommended in case of multiple abscesses. In case patients present with signs of sepsis or septic shock, laparoscopic drainage can be an alternative for percutaneous drainage, as promising results have been reported for this treatment strategy (15).

**FIGURE 2:**
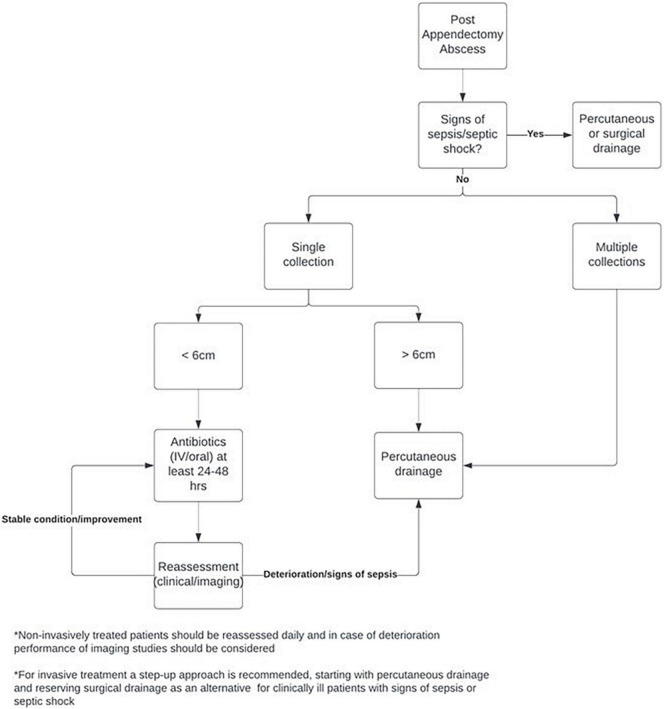
Standardized treatment protocol.

Based on the results of this study a standardized treatment protocol was developed, which is displayed in Figure 2. The treatment protocol is based on a step-up approach, especially for PAA smaller than 6 cm. After diagnosis of the PAA, the treating surgeon should firstly assess the patient for any signs of sepsis or even septic shock. In these patients, prompt evacuation of the PAA with percutaneous or surgical drainage procedures is needed. If the patient is in a stable condition without signs of sepsis or septic shock, treatment depends on the number of collections. As non-invasive treatment has been found to be less suitable and effective for multiple PAAs, invasive drainage procedures are recommended for these patients. If only a single PAA is found on imaging, treatment depends on abscess size. Based on expert opinion and an expected shorter length of hospital stay, percutaneous drainage of large (>6 cm) PAA is recommended. For the group of patients with a small to moderate size PAA (<6 cm), a step-up approach starting with non-invasive treatment with IV or oral antibiotics (or no antibiotics in specific cases) for at least 24–48 h is advised. If the patient is in a stable condition and PAA symptoms and/or size are improving, antibiotics can be continued. If the clinical condition of the patient deteriorates and/or signs of sepsis are present, the treating surgeon can proceed to percutaneous or surgical drainage. The decision to perform additional imaging studies should be based on the clinical condition of the patient.

The results of our study should be considered in the light of some limitations. First, the non-invasive and invasive treatment groups should be compared with caution due to confounding by indication. For all patients decisions for non-invasive and invasive treatment strategies were made by the treating surgeon. These decisions were influenced by several factors, such as the availability of a skilled (pediatric) interventional radiologist and the feasibility to perform percutaneous drainage. Due to the retrospective nature of this study, reasons for treatment decisions are limitedly described. Furthermore, due to the retrospective design, the study is prone to information bias. Major strength, however, is the multicenter design with participation of a tertiary referral center, one peripheral teaching hospital with a preference for non-invasive treatment and another peripheral teaching hospital with a preference for invasive treatment of PAA. The different philosophies of the two peripheral teaching hospitals reduce the bias by indication, which improves comparability of the treatment groups. Furthermore, because of the multicenter design, this historical cohort study describes one of the largest series of children treated for PAA.

It can be concluded that non-invasive treatment of small and medium size (<6 cm) PAA in children seems to be a safe and effective treatment strategy. Based on the results of the systematic review and our multicenter cohort study, a standardized treatment protocol was developed. This treatment protocol recommends a step-up approach for children with a PAA <6 cm, reserving invasive drainage procedures for children in an unstable condition with signs of sepsis or septic shock. For children presenting with large (>6 cm) PAA or multiple PAAs, initial percutaneous drainage is advised. Current evidence, however, is limited to historical cohort studies, in which risk of bias is high due to confounding by indication. Therefore, prospective validation of our proposed treatment protocol is needed.

## Data Availability Statement

Requests for data sharing will be considered upon written request to the corresponding author. Deidentified participant data will be made available after receipt of a written proposal and a signed data sharing agreement.

## Ethics Statement

The studies involving human participants were reviewed and approved by the Medical Ethics Review Committee of the Amsterdam UMC, Medical Ethics Review Committee of the Northwest Hospital, and Medical Ethics Review Committee of the Juliana Children’s Hospital/Haga-Hospital. Written informed consent from the participants’ legal guardian/next of kin was not required to participate in this study in accordance with the national legislation and the institutional requirements.

## Author Contributions

PA, S-MT, IM, RB, JD, JS, MK, GZ, JA, TB, LH, and RG contributed to the conception and design the study. PA, S-MT, and IM were involved in data collection and analysis. RV was responsible for the systematic review searches in the bibliographic databases PubMed and Embase. All authors were involved in the design and development of the study protocol, involved in writing, and revision of the manuscript.

## Conflict of Interest

The authors declare that the research was conducted in the absence of any commercial or financial relationships that could be construed as a potential conflict of interest.

## Publisher’s Note

All claims expressed in this article are solely those of the authors and do not necessarily represent those of their affiliated organizations, or those of the publisher, the editors and the reviewers. Any product that may be evaluated in this article, or claim that may be made by its manufacturer, is not guaranteed or endorsed by the publisher.
