# Supplementary material for: The Management of Post-appendectomy Abscess in Children; A Historical Cohort Study and Update of the Literature
Source: Front Pediatr. 2022 Jun 20;10:908485. doi: 10.3389/fped.2022.908485 (PMC9254404; doi:10.3389/fped.2022.908485)
Supplement: Supplementary file 1 [file Table_1.DOCX]

**Appendix 1. Search strategy**

The Pubmed search existed of the following combination of terms:

(("Abscess"[Mesh] OR abscess*[tiab] AND "Appendix/surgery"[Mesh] OR "Appendix/therapy"[Mesh] OR "Appendicitis/surgery"[Mesh] OR "Appendicitis/therapy"[Mesh] OR "Appendicitis/complications"[Mesh] OR "Appendectomy"[Mesh] OR appendectomy[tiab] OR appendicectomy[tiab] OR postappendectomy[tiab] OR postappendicectomy[tiab] AND child*[tw] OR schoolchild*[tw] OR infan*[tw] OR adolescen*[tw] OR pediatri*[tw] OR paediatr*[tw] OR neonat*[tw] OR boy[tw] OR boys[tw] OR boyhood[tw] OR girl[tw] OR girls[tw] OR girlhood[tw] OR youth[tw] OR youths[tw] OR baby[tw] OR babies[tw] OR toddler*[tw] OR teen[tw] OR teens[tw] OR teenager*[tw] OR newborn*[tw] OR postneonat*[tw] OR postnat*[tw] OR perinat*[tw] OR puberty[tw] OR preschool*[tw] OR suckling*[tw] OR picu[tw] OR nicu[tw])

The EMBASE search existed of the following combination of terms:

((('appendix'/exp AND ('surgery'/lnk OR 'complication'/lnk OR 'therapy'/lnk)) OR ('appendicitis'/exp AND ('surgery'/lnk OR 'complication'/lnk OR 'therapy'/lnk)) OR 'appendectomy'/exp OR 'appendectomy complications'/exp OR appendectomy:ab,ti,kw OR appendicectomy:ab,ti,kw OR postappendectomy:ab,ti,kw OR postappendicectomy:ab,ti,kw AND 'abscess'/exp OR abscess*:ab,ti,kw AND 'child'/exp OR 'adolescent'/exp OR child*:ab,ti,kw OR schoolchild*:ab,ti,kw OR infan*:ab,ti,kw OR adolescen*:ab,ti,kw OR pediatri*:ab,ti,kw OR paediatr*:ab,ti,kw OR neonat*:ab,ti,kw OR boy:ab,ti,kw OR boys:ab,ti,kw OR boyhood:ab,ti,kw OR girl:ab,ti,kw OR girls:ab,ti,kw OR girlhood:ab,ti,kw OR youth:ab,ti,kw OR youths:ab,ti,kw OR baby:ab,ti,kw OR babies:ab,ti,kw OR toddler*:ab,ti,kw OR teen:ab,ti,kw OR teens:ab,ti,kw OR teenager*:ab,ti,kw OR newborn*:ab,ti,kw OR postneonat*:ab,ti,kw OR postnat*:ab,ti,kw OR perinat*:ab,ti,kw OR puberty:ab,ti,kw OR preschool*:ab,ti,kw OR suckling*:ab,ti,kw OR picu:ab,ti,kw OR nicu:ab,ti,kw
