# Supplementary material for: The Management of Post-appendectomy Abscess in Children; A Historical Cohort Study and Update of the Literature
Source: Front Pediatr. 2022 Jun 20;10:908485. doi: 10.3389/fped.2022.908485 (PMC9254404; doi:10.3389/fped.2022.908485)
Supplement: Supplementary file 2 [file Table_2.DOCX]

**Appendix 2. Definitions**

Appendicitis severity: The severity of appendicitis was classified as simple or complex appendicitis, based on the perioperative and histopathological classification as described by Bhangu. [23]

Simple appendicitis: The macroscopic appearance of an increased diameter of the appendix, without signs of necrosis or perforation.

Complex appendicitis: Appendicitis with signs of necrosis (gangrenous appendicitis) or perforation, or the presence of an appendiceal abscess.

Recurrent/persistent abscess: A radiologically confirmed abscess on the same or a new location, that requires additional intervention.

Surgical site infection (SSI): Superficial and deep SSI were defined according to the CDC criteria. Superficial SSI was defined as inflammation of the skin and subcutaneous tissue. Deep SSI was defined as inflammation of deep soft tissues of the incision, such as the muscles, fascia or surrounding tissues. [24]

Fistula: A clinically and/or radiologically confirmed passage between intra-abdominal organs or an intra-abdominal organ and the body surface.

Small bowel obstruction: The diagnosis of (adhesive) small bowel obstruction was based on clinical signs and symptoms such as history of constipation, nausea, vomiting, and distended abdomen.
